# Vaccination of cattle with synthetic peptides corresponding to predicted extracellular domains of *Rhipicephalus (Boophilus) microplus* aquaporin 2 reduced the number of ticks feeding to repletion

**DOI:** 10.1186/s13071-022-05166-1

**Published:** 2022-02-08

**Authors:** Glen A. Scoles, Hala E. Hussein, Cassandra L. Olds, Kathleen L. Mason, Sara K. Davis

**Affiliations:** 1 USDA-ARS, Animal Disease Research Unit, Washington State University, Pullman, WA USA; 2 Department of Veterinary Microbiology and Pathology, Washington State University, Pullman, WA USA; 3 Department of Entomology, Faculty of Science, Cairo University, Giza, Egypt; 4 Department of Entomology, Plant Pathology and Nematology, University of Idaho, Moscow, ID USA; 5 Present Address: USDA-ARS, Invasive Insect Biocontrol and Behavior Lab, Beltsville Agricultural Research Center, Beltsville, MD USA; 6 Present Address: Department of Entomology, Kansas State University, Manhattan, KS USA

**Keywords:** Aquaporin, Cattle tick, Bm86, IgG isotype, Anti-tick vaccine

## Abstract

**Background:**

There have been ongoing efforts to identify anti-tick vaccine targets to protect cattle from infestation with cattle fever ticks *Rhipicephalus* (*Boophilus) microplus*. Two commercial vaccines based on the tick gut protein Bm86 have had variable effectiveness, which has led to poor acceptance, and numerous studies have attempted to identify vaccine antigens that will provide more consistently effective protection. Transcriptomic analysis of *R. microplus* led to identification of three aquaporin genes annotated to code for transmembrane proteins involved in the transport of water across cell membranes. Previous work showed that vaccination with full-length recombinant aquaporin 1 (RmAQP1) reduced tick burdens on cattle. Targeted silencing of aquaporin 2 (RmAQP2) expression suggested it might also be a good anti-tick vaccination target.

**Methods:**

Three synthetic peptides from the predicted extracellular domains of RmAQP2 were used to vaccinate cattle. Peptides were conjugated to keyhole limpet hemocyanin (KLH) as an antigenic carrier molecule. We monitored the antibody response with ELISA and challenged vaccinated cattle with *R. microplus* larvae.

**Results:**

There was a 25% reduction overall in the numbers of ticks feeding to repletion on the vaccinated cattle. Immune sera from vaccinated cattle recognized native tick proteins on a western blot and reacted to the three individual synthetic peptides in an ELISA. The vaccinated calf with the highest total IgG titer was not the most effective at controlling ticks; ratios of IgG isotypes 1 and 2 differed greatly among the three vaccinated cattle; the calf with the highest IgG1/IgG2 ratio had the fewest ticks. Ticks on vaccinated cattle had significantly greater replete weights compared to ticks on controls, mirroring results seen with RNA silencing of RmAQP2. However, protein data could not confirm that vaccination had any impact on the ability of the tick to concentrate its blood meal by removing water.

**Conclusions:**

A reduced number of ticks feed successfully on cattle vaccinated to produce antibodies against the extracellular domains of RmAQP2. However, our predicted mechanism, that antibody binding blocks the ability of RmAQP2 to move water out of the blood meal, could not be confirmed. Further study will be required to define the mechanism of action and to determine whether these vaccine targets will be useful components of an anti-tick vaccine cocktail.

**Graphical Abstract:**

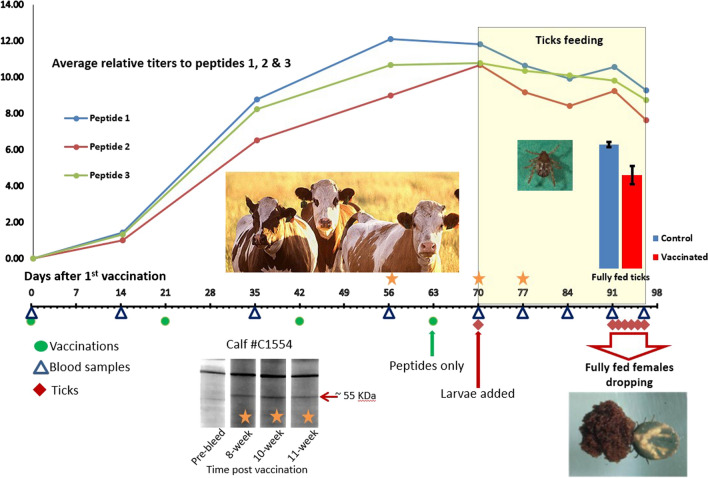

**Supplementary Information:**

The online version contains supplementary material available at 10.1186/s13071-022-05166-1.

## Background

The cattle tick *Rhipicephalus* (*Boophilus*) *microplus* causes both direct and indirect injury to livestock worldwide. Direct injury due to tick feeding results in significant production losses and damage to hides. Indirectly, *Boophilus* ticks are vectors of several globally important pathogens causing anaplasmosis and babesiosis, which result in significant morbidity and mortality [1]. Acaricide use is the most common means of tick control to prevent both direct and indirect injury; however, acaricides are expensive, can result in residues in meat and dairy products, may cause environmental contamination, and resistance has developed to several classes of acaricides [2]. It has been suggested that vaccines would be the most effective and environmentally sound approach for the prevention and control of ticks and tick-borne pathogens [3]. Early development of the concept of controlling ticks by vaccination centered on attempting to understand the phenomenon of naturally acquired anti-tick immunity [4]. However, naturally acquired immunity is not sufficient to prevent damage and disease transmission, and the idea of targeting concealed antigens as anti-tick vaccines was proposed [5]. The observation that vaccination of cattle with the concealed “*Boophilus microplus”* tick midgut antigen Bm86 could reduce tick burdens led to efforts to develop commercial anti-tick vaccines. Although *Boophilus microplus* has been reclassified as *Rhipicephalus microplus* [6], the Bm86 protein has retained the original “Bm” designation. The first Bm86 vaccine, TickGARD™ (Hoechst Animal Health; Australia), was developed and marketed in Australia [7], and later Gavac™ (Heber Biotec; Havana, Cuba), also based on Bm86, was developed in Cuba [8] and marketed in Latin America [9]. However, neither of these vaccines has been a sustained commercial success. TickGARD™ is no longer on the market and Gavac™ has limited availability. The limited commercial success of vaccines based on Bm86 was primarily due to market considerations driven by variable effectiveness against different tick populations, and the need for frequent boosts to maintain effective levels of immunity [10]. Because these commercially available vaccines reduce, but do not eliminate, the need for acaricides, they were intended to be incorporated into an integrated management strategy which also includes the use of acaricide, albeit at a reduced frequency [1, 9]. The development and use of a new generation of anti-tick vaccines is an emerging alternative means for tick control [3, 11–14]. Research efforts are ongoing to identify anti-tick vaccine targets that will be more consistently effective than Bm86 has been [14], and several tick antigens including *R. microplus* glutathione-S transferase, ubiquitin, selenoprotein W, elongation factor 1-alpha, aquaporins, subolesin, and others have been proposed as potential vaccine candidates for use alone or in combination with Bm86 [3, 14].

Aquaporins are a family of integral transmembrane proteins that are broadly conserved across taxa. The proteins that make up the transmembrane aquaporin channel are responsible for active movement of water and solutes across cell membranes [15]. Ticks concentrate their blood meal by actively moving water out of the gut and returning it to the host via saliva [16] using active transport mechanisms such as aquaporins. Aquaporins have been proposed as good anti-tick vaccine candidates because water balance is a critical biological activity for blood-feeding ticks and because there are exposed extracellular domains on aquaporin proteins that could be easily targeted to block water channel function. Hypothetically, antibody binding to extracellular domains of aquaporins in the tick gut and salivary glands could abrogate aquaporin function, reducing the ability of the tick to actively concentrate the blood meal and reducing the volume of saliva the tick can inject back into the host. This should lower the protein content of the blood meal, which would reduce fecundity and could also interfere with interactions at the host–parasite interface that are mediated by saliva, such as immunosuppression, histamine binding, and anticoagulation [17, 18].

Three aquaporin cDNAs have been identified in transcriptomic studies of *R. microplus* [19, 20]. When aquaporin 1 (RmAQP1) was expressed as a full-length protein and tested in a vaccine trial, it resulted in significant protection against feeding ticks [21]. Targeted gene silencing studies with aquaporin 2 (RmAQP2) [22] resulted in reduced tick survival and increased replete weights, suggesting that ticks were less able to remove excess water from the blood meal [22]. Vaccination targeting aquaporin proteins of other tick species has shown similar effects [23, 24].

Structural modeling suggests that RmAQP2 has surface-exposed extracellular domains that contain predicted B-cell epitopes [22]. In the current study we tested the hypothesis that vaccination of cattle with synthetic peptides designed from expressed sequences representing these extracellular domains would induce production of antibodies that reduce tick feeding success and fecundity by interfering with the critically important biological functions of aquaporin in the tick.

## Methods

### Cattle and ticks

All animal use was approved by the University of Idaho Institutional Animal Care and Use Committee (IACUC, protocol #2016–27). Six age-matched male Holstein calves were acquired from the University of Idaho dairy. Calves were ≥ 2 months of age with ≥ 80 kg body weight at the start of the study. This cohort of cattle had no previous exposure to *R. microplus*, since the tick does not occur in the United States north of the quarantine zone along the border with Mexico. Animals were randomly assigned to either the control or vaccine group (three per group) and vaccinated according to the protocol described below. At the end of the vaccination protocol, cattle were moved to individual moated concrete block stalls for the tick challenge. For the tick challenge (see below) the La Minita strain of *R. microplus* was used. This tick colony originated from an outbreak tick population in Star County, Texas [25, 26], and has been maintained continuously (3–4 generations per year) at the Animal Disease Research Unit tick lab at the University of Idaho since it was acquired from Texas.

### Peptides

Three peptide sequences from the predicted extracellular domains of the protein coded for by the RmAQP2 gene (GenBank accession numbers: protein ALJ75650, DNA Sequence KP406519) have been described previously [22]. Peptide 1 was modified from the previously published sequence by adding four additional amino acids to encompass a predicted B-cell epitope and possibly increase its antigenic potential (AVFQLGSVGLAAAP). The amino acid sequences of peptides 2 and 3 were as described previously (#2: ADALSQVDVNLAIVYGTNATAPVFSCFPAPGV, #3: MCGWGSAVFSFRSYNWFWV) [22]. Peptides were commercially synthesized (New England Peptide, Gardner, MA, USA) and supplied either as free peptide or conjugated to carrier molecules. For vaccination, peptides were conjugated to keyhole limpet hemocyanin (KLH) as a carrier to stimulate immune response. For use in enzyme-linked immunoassay (ELISA; see below) peptides were conjugated to bovine serum albumin (BSA). KLH-conjugated peptides were supplied lyophilized and were initially dissolved by adding 50:50 dimethyl sulfoxide (DMSO)/phosphate-buffered saline (PBS), then taken to 1 mg/ml concentration with PBS for a final DMSO concentration of 20%. Unconjugated peptides were solubilized by adding 75:25 DMSO/sterile water and then taken to a 1 mg/ml concentration with PBS for a final DMSO concentration of 35%. BSA-conjugated peptides were obtained solubilized in PBS at 1 mg/ml concentration.

### Vaccination protocol

**Fig. 1:**
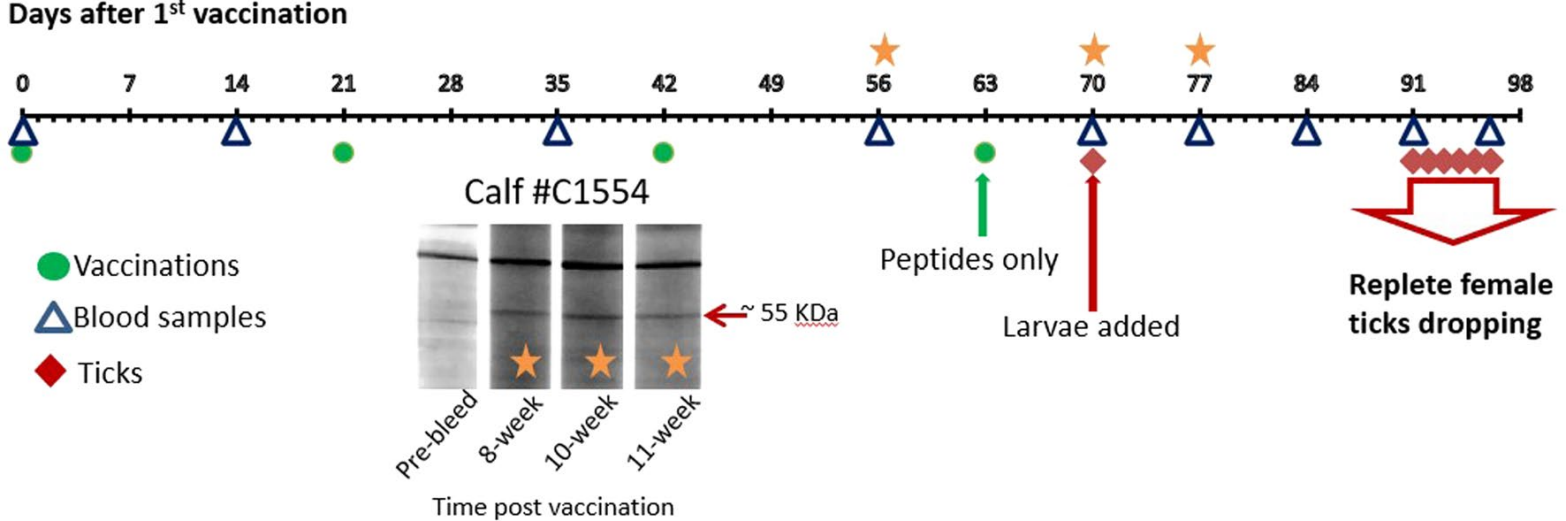
Timeline of experiment and western blot showing immune response to native tick protein after vaccination with peptides. Timeline represents days, counted from the first vaccination. Green dots represent vaccinations, with the first three vaccinations (days 0, 21, and 42) done at 3-week intervals using peptides conjugated to KLH, and the fourth vaccination (day 63) consisting of peptides alone. Blue triangles indicate blood samples taken before the initial vaccination (pre-bleed), 2 weeks after each vaccination, and weekly during the tick feed (days 0, 14, 35, 56, 70, 77, 84, 91, 98). Red diamonds indicate dates ticks were placed on (day 70) and collected as detached repletes (days 91–96). Orange stars on the timeline correspond to lanes on the western blot showing reactivity of serum from representative peptide-vaccinated calf C1554, with native tick protein showing reactivity to a 50–55 KDa band in this calf; the other two vaccinated calves showed similar reactivity

Cattle were each vaccinated four times at 3-week intervals (days 0, 21, 42, and 63); see Fig. 1 for a graphical representation of the experimental timeline. The initial three vaccinations were done with the peptides conjugated to KLH; the fourth injection (day 63) was with peptides alone, not conjugated to KLH, in order to boost the peptide-specific immune response in the absence of KLH. Each injection consisted of 0.05 mg of a single conjugated peptide and 0.75 mg Quil-A (saponin) adjuvant in a volume of 0.5 ml. On the day of vaccination, each animal received three injections, each with a different peptide, at three different injection sites. Individual peptides were mixed with adjuvant by drawing repeatedly through a 21-gauge needle to mix well. All three doses of each peptide were mixed in a single tube, allowing an extra dose for loss in mixing and in the needle (1 ml 0.2 mg/ml peptide plus 1 ml 3 mg/ml Quil-A). Each injection was administered subcutaneously at a different injection site to avoid any potential antigenic competition at the draining lymph node (injection sites were left neck, right neck, right flank). The controls were injected with saponin mixed with KLH (MilliporeSigma, St. Louis, MO, USA) alone (no conjugated peptide), in the same way as the KLH conjugated peptides were prepared and injected (i.e., three injections at three different sites).

### Tick challenge

One week after the final peptide boost, the cattle were challenged with larval ticks. A tick-feeding patch was adhered to the back of each calf with cattle hip tag cement as described previously [27], and each patch was infested with ≈5000 larval ticks, which is the approximate number of larvae hatching from 0.25 g of eggs. Larval ticks were prepared for challenge by weighing aliquots of 0.25 g of eggs all originating from the same colony rearing; the eggs were from a mixture of egg masses from a large number of females which had all been mixed and weighed on the same day to ensure uniformity. Because *R. microplus* is a one-host tick, larvae will develop through the larval, nymphal, and adult stages on the same host. Feeding patches were opened on days 5 and 9 post-application to assess attached larvae and newly molted nymphs, respectively, as they progressed through stages of feeding and development. After replete females began to detach at about day 20, the patches were opened daily, and all repletes that had dropped in the previous 24-h period were removed. Daily collections were repeated until most ticks had completed feeding; on the final day all remaining attached ticks were removed and counted.

The total number of detached replete (fully fed) ticks collected each day was recorded. A random sample of up to 72 ticks each day were weighed and saved in individual wells of 24-well tissue culture plates. On days when there were fewer than 72 ticks, all were weighed and saved. Saved ticks were held for egg production, and when all oviposition was complete, egg mass weights were recorded. A subsample of eggs from up to 48 of these ticks per day was set aside individually to assess hatching rate. A daily sample of 10 replete females from each calf was saved for protein determination (see below). All remaining ticks were discarded after they were counted.

### Antibody titers

To assess antibody titers and to determine what titers the ticks were actually exposed to, blood samples were taken from cattle before the first vaccination (pre-bleed) and 2 weeks after each subsequent vaccination, then weekly once the tick challenge began (days 0, 14, 35, 56, 70, 77, 84, 91, and 96). Blood was collected in red-top Vacutainer tubes and allowed to clot before being centrifuged to separate serum. An ELISA was developed to track antibody titers in response to vaccination. Nunc Polysorp 96-well flat-bottom immuno-plates (Thermo Scientific, Rochester, NY, USA) were coated with BSA-conjugated peptides. Each BSA-conjugated peptide was diluted to 0.02 mg/ml in carbonate-bicarbonate coating buffer (4 mM Na2CO3, 9 mM NaHCO3, pH 9.4); 50 µl of this peptide working solution was added to each well and held overnight at 4 °C to coat the wells with approximately 1 µg/well of peptide available to bind. Plates were washed five times by hand with 1× PBS/0.05% Tween-20 to remove unbound peptide after coating, then blocked with 225 µl/well of 1× PBS/0.05% Tween-20 plus 5% BSA (blocking buffer) for 2 h.

Diluted samples (50 µl) were added to the plates in triplicate wells and allowed to incubate for 1 h at room temperature. Unbound primary antibody was removed by washing five times by hand with 1× PBS/0.05% Tween-20, and 50 µl/well of a 1:500 dilution (in blocking buffer) of goat anti-bovine IgG (H+L) antibody-horseradish peroxidase (HRP) conjugate (Life Technologies, Frederick, MD, USA) was applied for 1 h at room temperature. Unbound secondary antibody was removed by washing five times by hand with 1× PBS/0.05% Tween-20. Plates were developed with SigmaFast OPD (MilliporeSigma, St. Louis, MO, USA) and read at 450 nm on a SpectraMax 190 microplate reader (Molecular Devices, San Jose, CA, USA).

Using the ELISA described above, we determined that a 1:256 dilution was optimal for use across all the available sample time points to show the change in antibody levels during the study. To determine the peak titers, the sample point with the highest antibody response at 1:256 for each peptide was chosen, and twofold serial dilution series of the serum samples from this day were tested to find the lowest dilution where the 95% confidence intervals for the pre-bleed and the test sample did not overlap. The reciprocal of the dilution at this point was considered to be the maximum antibody titer.

### Measurement of bovine IgG1 and IgG2 using ELISA

Isotyping was completed similarly to the protocol above, with the difference that the secondary antibodies were un-conjugated and an additional HRP conjugate was used for detection. Specifically, the secondary antibodies were mouse anti-bovine IgG1 or mouse anti-bovine IgG2 (Bio-Rad Laboratories, Hercules, CA, USA) and the HRP conjugate was goat anti-mouse IgG (H+L) (Life Technologies, Frederick, MD, USA).

### Protein content of replete female ticks

A random sample of 10 replete females were collected from each calf on each day after the ticks began dropping for a total of 6 days (60 ticks) from each calf. After weighing, replete female ticks were placed individually in tubes with 2 ml protein lysis buffer solution containing 0.05 M Tris, 0.005 M EDTA, 1% NP-40, and protease inhibitors (cOmplete™, Mini, EDTA-free Protease Inhibitor Cocktail, Roche, MO, USA), and frozen at −20 °C. In preparation for the assay, tubes were thawed, the tick bodies were punctured with a 16-gauge needle, and the tissues and solution were drawn into an attached 3-ml syringe approximately 15 times to thoroughly homogenize each sample. An aliquot of 200 μl was taken from each sample and centrifuged at 8000 rpm to pellet unlysed material (cuticle, tissue fragments, etc.), and 10 μl of the supernatant was diluted in 490 μl sterile water. Triplicate samples of 25 μl of each dilution were analyzed for total protein concentration using the Pierce BCA Protein Assay according to the manufacturer’s protocol (Thermo Scientific). A 1:50 dilution of protein lysis buffer above with sterile water was used as a diluent for the BSA standard curve. Completed plates were incubated at 37 °C for 30 min and read at 562 nm on a SpectraMax 190 microplate reader (Molecular Devices, San Jose, CA, USA). The basis for comparison between populations of ticks from vaccinated versus control cattle was total soluble protein as measured by the assay as a proportion of total tick weight.

### Western blots

Ovaries from fed female *R. microplus* (3–4 days and replete) were dissected, placed in RNAlater solution (Thermo Fisher Scientific, Waltham, MA, USA), and stored at −80 °C. To prepare protein for the western blot, samples were thawed and RNAlater was carefully pipetted off the tissues. Protein lysis buffer (0.05 M Tris, 0.005 M EDTA, 1% NP-40, cOmplete™ mini EDTA-free protease inhibitor cocktail; Roche, MO, USA) was added to the tissues according to the manufacturer’s instructions. Tubes were sonicated using a cup horn (Fisherbrand Model 705, Thermo Fisher Scientific) for 6 × 15 s at 100% power and cooled on ice between steps. The prepared samples were then analyzed using a Qubit 3 Fluorometer (Thermo Fisher Scientific) to determine protein concentration, and 25 μl of a 1:10 dilution of replete female ovary protein was prepared to run on a NuPage 4–12% Bis–Tris gel (1.0 mm × 10 wells, Thermo Fisher Scientific) by adding 12.5 μl NuPage 4× LDS loading buffer, 5 μl NuPage 10× sample reducing agent, and 7.5 μl sterile water. The mixture was vortexed and heated in a dry block at 70 °C for 10 min. A 20-gauge ½-inch needle was used to carefully remove pre-cast lane dividers between four wells of the gel to form one large well. The gel was then secured in the Mini Gel Tank (Thermo Fisher), 1× NuPage MOPS buffer containing antioxidant was added to the inner chamber, and the same buffer without antioxidant was added to the outer chamber. Ovary protein was pipetted into the large open well of the gel, and a mixture of 2.5 μl PageRuler™ Plus pre-stained and 2.5 μl MagicMark™ XP western ladder (both Thermo Fisher Scientific) was pipetted into a separate adjoining single well. Electrophoresis was completed for ~ 50 min at a constant 200 V.

The proteins were then transferred to a nitrocellulose membrane using the iBlot gel transfer stack (Thermo Fisher Scientific) and Program PO (20 V for 1 min, 23 V for 4 min, 25 V for 2 min.) The membrane was blocked with 1× Tris-buffered saline (TBS) + 0.1% Tween-20 + 5% non-fat milk for 1 h at room temperature. Equal strips were cut from the blocked membrane, serum from calves was diluted 1:1 in 1× TBS + 0.1% Tween-20 + 10% non-fat milk and incubated with the membrane strips for 2 h at room temperature, rocking side to side gently. After primary incubation was completed, the strips were washed in 1× TBS + 0.1% Tween-20 twice immediately and then three times for 5 min each. Goat anti-mouse IgG (H+L)-HRP antibody (Thermo Fisher Scientific) was diluted 1:5000 in 1× TBS + 0.1% Tween-20 + 5% non-fat milk for the ladder lane and control strip. Rabbit anti-bovine IgG (whole molecule)-HRP antibody (MilliporeSigma, St. Louis, MO, USA) was diluted 1:2000 in 1× TBS + 0.1% Tween-20 + 5% non-fat milk for the bovine serum strips. Membrane strips were incubated with the secondary antibody dilutions for 1 h at room temperature, rocking gently. After secondary incubation was completed, the strips were washed as described above. Prometheus ProSignal Pico (Genessee Scientific, San Diego, CA, USA) components were prepared 1:1 for imaging the blot. Enhanced luminol solution was added to stabilized peroxide solution, mixed, and placed on the washed strips for 2 min. Strips were drained of excess reagent, arranged in a blot development folder, and imaged using a ChemiDoc XRS System (Bio-Rad Laboratories, Hercules, CA, USA).

### Analysis of data

Tick data (number and weight of replete females, weight of egg masses, and egg hatching, average protein content, etc.) were collected daily and summarized as the average for that day for each calf. The daily averages were combined as a single value for each animal across the full 6 days the ticks were dropping, and the values for the three vaccinated and three control animals were compared using Student’s *t*-test (implemented in Microsoft Excel).

## Results

### Timeline for vaccination, serum sampling, and tick application

Figure 1 presents a timeline for the experiment including the days of vaccination (days 0, 21, 42, and 63), the days bovine serum samples were collected for determination of antibody titers (days 0, 14, 35, 56, 70, 77, 84, 91, and 96), and the days ticks were applied and collected (larvae applied day 70, replete ticks dropping days 91–96). All vaccinated and control cattle tolerated the vaccinations well and there were no adverse reactions at the injection sites; however, our animal health records from the experiment suggest that one of the vaccinated calves, C1551, suffered from failure to thrive and had lower weight gain than the other five calves. After the last day of tick drop, a final serum sample was collected and cattle were all euthanized (day 96).

### Bovine immune response to native tick protein

Antibody from the vaccinated cattle bound to tick protein isolated from the ovary of engorged female ticks on a western blot. Although serum from vaccinated and unvaccinated cattle bound to many different tick proteins on the blot, there was one clear ovary protein band at approximately 50–55 kDa that was bound only by serum from vaccinated cattle and was not bound by either pre-bleed serum or serum from control (unvaccinated) animals. All vaccinated cattle produced antibody that bound to this band; a representative blot for vaccinated calf C1554 is shown in Fig. 1.

### Antibody titers

**Fig. 2:**
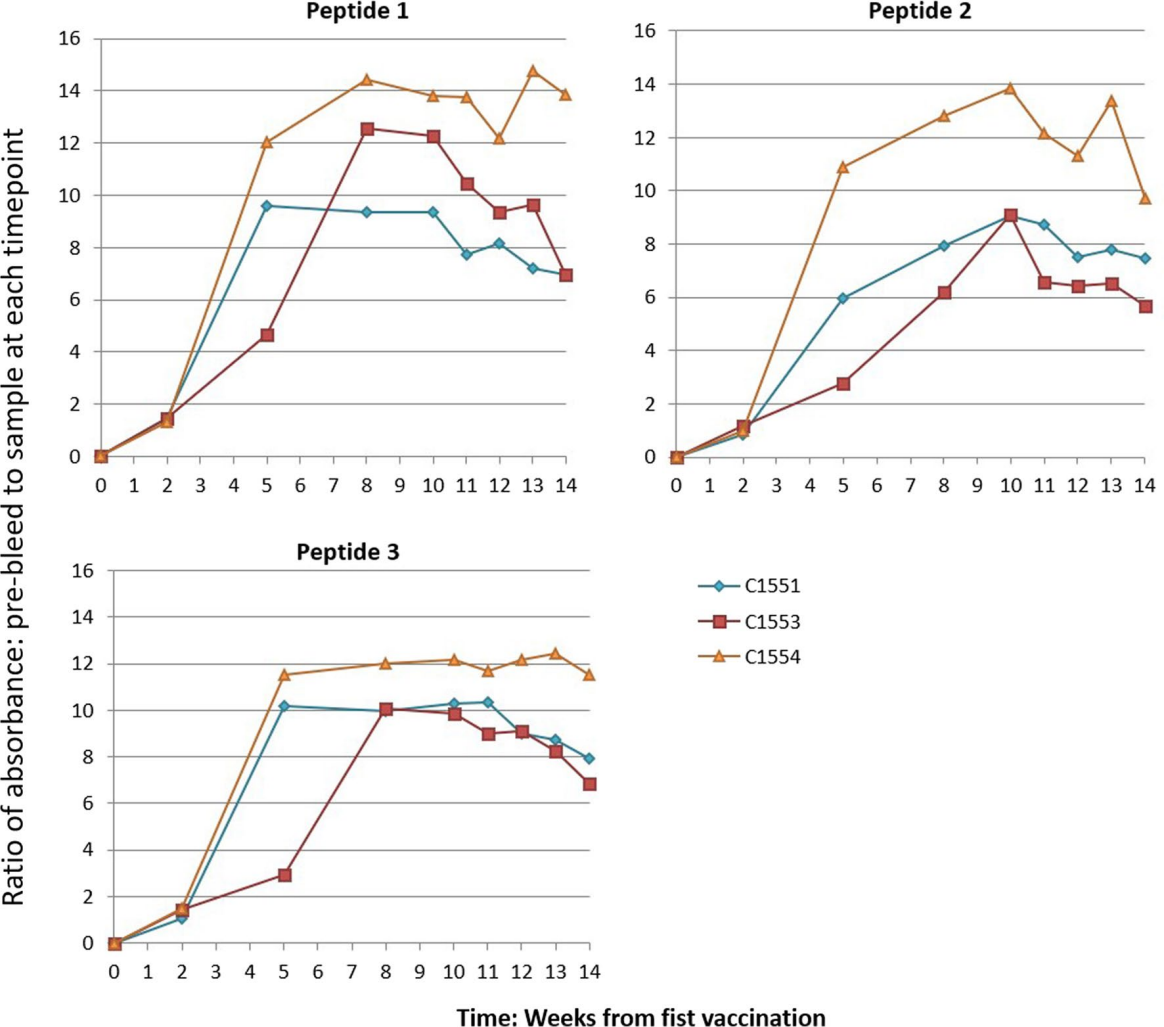
Antibody titers across the experimental time course. Total IgG response to vaccination for each aquaporin peptide (peptides conjugated to KLH). Shown as the ratio of the absorbance at 450 nm between the pre-bleed sample and the sample at each time point. Sample dilution 1:256. Refer to Fig. 1 for critical experimental time points (last vaccination at week 9, ticks dropping weeks 13–14)

**Fig. 3:**
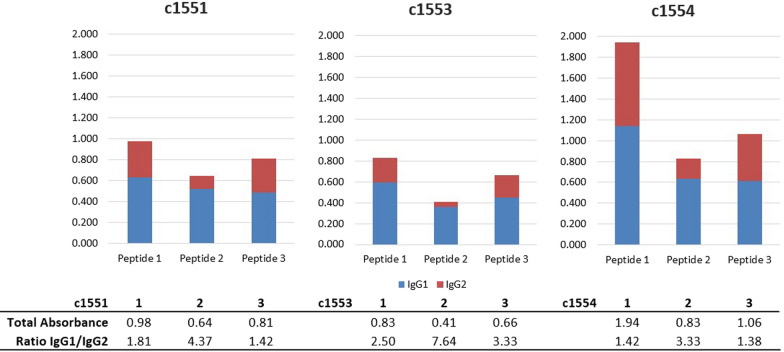
Antibody isotyping: stacked bar graph showing relative amount (absorbance at 450 nm) of IgG1 and IgG2 for each peptide from each calf. The ratio of absorbance of IgG1/IgG2 is shown below the graphs for each calf and each peptide

To compare the antibody titers of all samples over the course of the experiment from pre-bleed through the end of the tick feeding, all sera were tested in the ELISA using a serum dilution of 1:256 with blocking buffer (Fig. 2 and Additional file 1: Data file S1). To determine the peak titer for each calf against each peptide, we selected the day that the average response to the 1:256 dilution was the highest and tested a full twofold serial dilution series on serum from that sample point. The average titer at 1:256 was the highest at week 8 for peptide 1, week 10 for peptide 2, and week 8 for peptide 3. The inverse of the greatest dilution before all reactivity was lost was considered the maximum antibody titer (Table 1 and Additional file 2: Data file S2). The relative amounts of IgG isotypes IgG1 and IgG2 were determined for each peptide for each calf at the same time points as the maximum antibody titers (Table 2, Fig. 3, and Additional file 3: Data file S3). All cattle produced more IgG1 than IgG2 (ratio above 1.0). Vaccination with peptide 2 led to greater relative amounts of IgG1. Calf C1554 produced the greatest amount of total antibody (Fig. 2) but had the lowest ratios of IgG1/IgG2.

**Table 1:** Peak titers for each peptide

	Peptide 1	Peptide 2	Peptide 3
Week of sample	8	10	8
Calf number			
C1551	1024	1024	2048
C1553	512	512	1024
C1554	1024	1024	2048

Lowest dilution of a onefold serial dilution series before loss of reactivity to BSA-conjugated peptides in an ELISA

**Table 2:** Relative amounts of antibody isotypes IgG1 and IgG2

Calf number	Peptide	IgG1	IgG2	Total	IgG1/IgG2
C1551	1	0.630	0.349	0.979	1.81
	2	0.523	0.120	0.642	4.37
	3	0.486	0.328	0.814	1.48
C1553	1	0.593	0.237	0.830	2.50
	2	0.359	0.047	0.406	7.64
	3	0.448	0.215	0.663	2.08
C1554	1	1.138	0.803	1.941	1.42
	2	0.637	0.191	0.828	3.33
	3	0.616	0.448	1.064	1.38

Data from samples taken at the highest antibody titer (week 8 for peptides 1 and 3, week 10 for peptide 3). Dilution 1:512, values are absorbance at 450 nm in ELISA plate reader

### Tick challenge

The feeding patches were opened to observe the ticks inside of them on days 5 and 9 after application of the ticks to check for tick attachment and early feeding success. Although tick numbers could not be quantified at these time points, fewer ticks were visible on these days on all vaccinated cattle as compared to the unvaccinated control cattle.

**Fig. 4:**
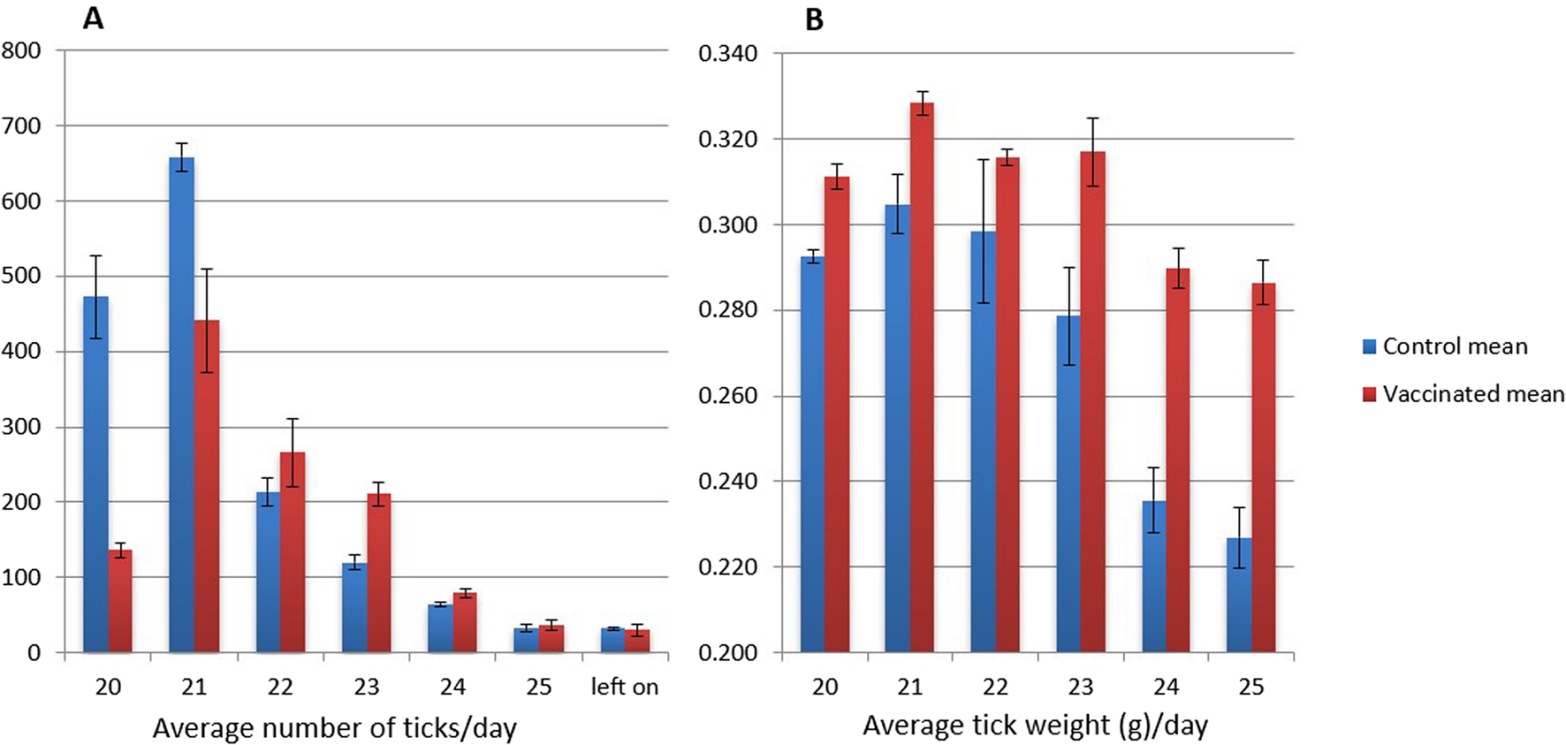
Bar graphs showing average tick numbers and weights for three control versus three vaccinated cattle over the 6 days replete ticks dropped, which were days 20–25 after the larvae were applied. **a** Average numbers of replete ticks dropping per calf per day; **b** average weight of replete ticks in grams per day. Ticks remaining attached on the 7th day were not weighed. Blue bars = control (unvaccinated), red bars = vaccinated. Error bars = standard error of the mean

Replete females had begun to detach on the 19th day after larvae were applied (corresponding to day 90 since the first vaccination); starting on tick day 20, the patches were opened daily and all ticks that had detached in the previous 24-h period were removed. All detached ticks were removed from the patches daily through tick day 25; on day 26 of tick feeding, all remaining attached and detached ticks were removed (corresponding to day 96 since the beginning of the experiment when the first vaccinations were given). Vaccinated cattle produced an average of 1201 (*N* = 3, SD = 199.8) ticks, as compared to an average of 1594 (*N* = 3, SD = 61.2) ticks for the control group, a statistically significant 24.7% reduction (one-tailed two-sample *t*-test, *df* = 4, *P* = 0.0156). The daily numbers of replete females and average tick weights for each day from each calf can be seen in Fig. 4 and in Tables 3 and 4; the complete data set for all individual ticks is available as Additional file 4: Data file S4.

**Table 3:** Total number of ticks collected per calf per day

	Day of collection							
	20	21	22	23	24	25	Left on	Total per calf
Control								
C1557	506	620	200	103	71	32	32	1564
C1559	364	672	251	136	61	40	29	1553
C1560	546	682	190	121	63	26	36	1664
Daily mean	472	658	214	120	65	33	32	**1594**
SD	95.65	33.29	32.72	16.52	5.29	7.02	3.51	61.16
SEM	55.22	19.22	18.89	9.54	3.06	4.06	2.03	35.31
Vaccinated								
C1551	130	574	348	228	74	39	32	1425
C1553	124	346	260	179	92	25	16	1042
C1554	156	403	190	225	72	46	43	1135
Daily mean	137	441	266	211	79	37	30	**1201**
SD	17.01	118.65	79.17	27.47	11.02	10.69	13.58	199.77
SEM	9.82	68.51	45.71	15.86	6.36	6.17	7.84	115.33

Day of collection = number of days since larval ticks were first applied. *SD* standard deviation, *SEM* standard error of the mean

**Table 4:** Average weight of ticks collected from each calf each day

	Day of collection												
	20		21		22		23		24		25		Mean per calf
	wt	*n*	wt	*n*	wt	*n*	wt	*n*	wt	*n*	wt	*n*	
Control													
C1557	0.292	72	0.294	72	0.278	72	0.260	72	0.230	59	0.213	22	0.269
C1559	0.291	72	0.318	72	0.332	72	0.299	72	0.251	51	0.237	29	0.296
C1560	0.295	72	0.303	72	0.285	72	0.276	72	0.226	53	0.231	16	0.278
Daily mean	0.293		0.305		0.298		0.279		0.236		0.227		0.281
SD	0.0024		0.0121		0.0292		0.0198		0.0130		0.0125		0.014
SEM	0.0014		0.0070		0.0168		0.0114		0.0075		0.0072		0.008
Vaccinated													
C1551	0.312	72	0.325	72	0.3150	72	0.312	72	0.283	62	0.293	29	0.309
C1553	0.306	72	0.326	72	0.3128	72	0.307	72	0.288	71	0.290	15	0.307
C1554	0.316	72	0.334	72	0.3190	72	0.332	72	0.298	62	0.276	35	0.316
Daily mean	0.311		0.328		0.3156		0.317		0.290		0.286		0.311
SD	0.0049		0.0050		0.0031		0.0135		0.0079		0.0088		0.0050
SEM	0.0028		0.0029		0.0018		0.0078		0.0046		0.0051		0.0029

wt = Average weight in grams for *n* ticks collected on each day. Day of collection = number of days since larval ticks were first applied. *n* = number of ticks include in the average for the day. *SD* standard deviation. *SEM* standard error of the mean

The average weight of replete ticks from vaccinated cattle was significantly greater than ticks from the control cattle, *M* = 0.311 g (*N* = 3, SD = 0.005) vs. *M* = 0.281 g (*N* = 3, SD = 0.014), respectively (one-tailed two-sample *t*-test, *df* = 4, *P* = 0.0012), a 10.7% increase (Table 4 and Fig. 4). The average difference in weight between the two groups increased over the time that fully fed adult ticks were dropping; ticks that took a longer time to feed to repletion showed a greater difference in weight between the vaccinated and unvaccinated groups (Table 4). Ticks collected after the first day of drop (tick day 20) from vaccinated cattle averaged 6.36% heavier than ticks from the control cattle, whereas by the sixth day of drop, ticks from vaccinated cattle averaged 26.25% heavier than ticks from the control (Table 4).

**Fig. 5:**
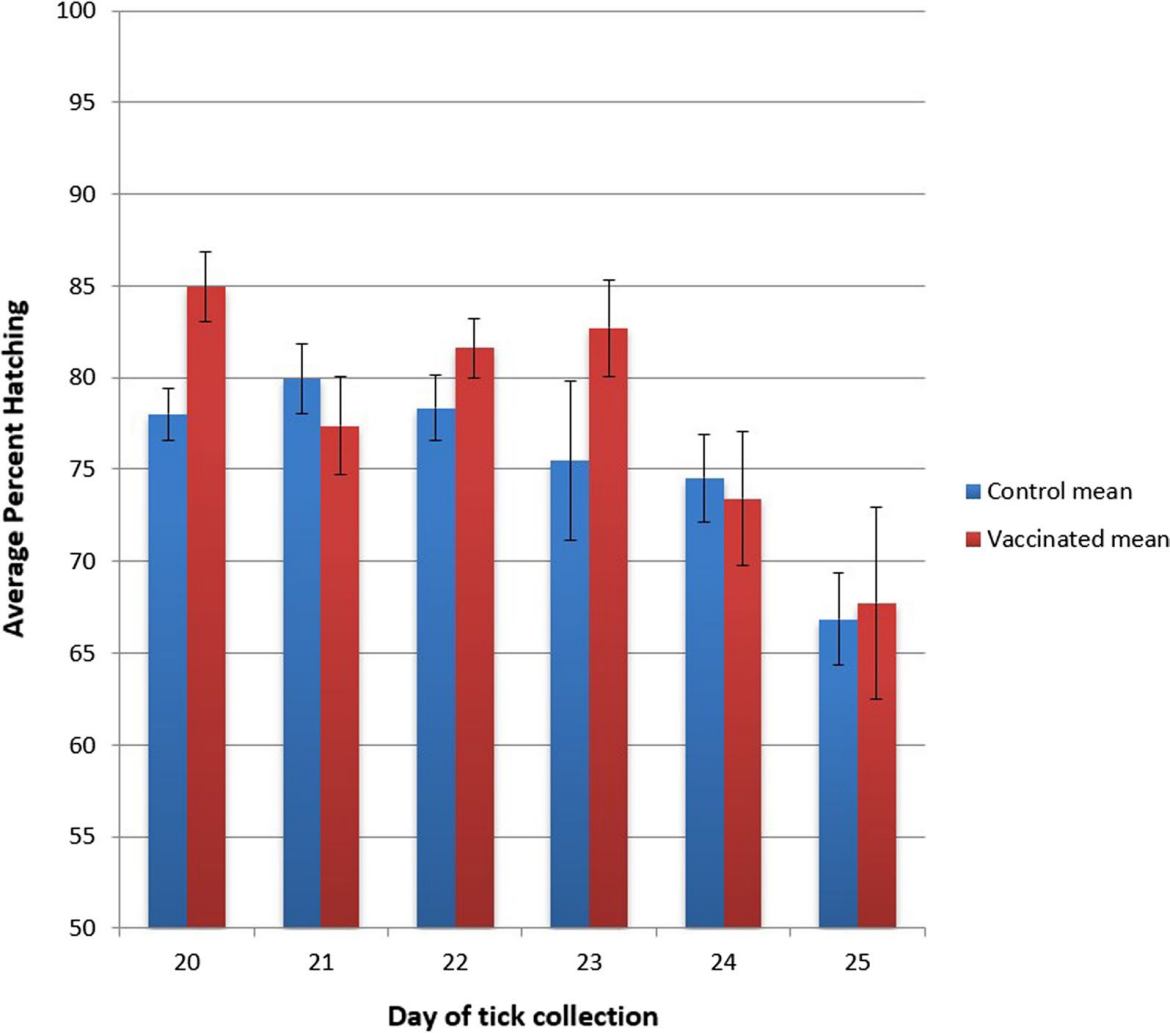
Daily egg hatching rate over 6 days ticks were dropping, which were days 20–25 after the larvae were applied. Average % hatching of a subsample of eggs from 48 egg masses from each calf on each day. Error bars = standard error of the mean. Blue bars = control (unvaccinated), red bars = vaccinated

Overall, ticks from vaccinated cattle produced a significantly greater weight of eggs than ticks from unvaccinated cattle (*M* = 0.160 g, *N* = 3, SD = 0.002 vs. *M* = 0.136 g, *N* = 3, SD = 0.007, respectively; one-tailed two-sample *t*-test, *df* = 4, *P* = 0.0028) (Table 5), and although the difference in conversion ratio was small, the percent of total body mass that was converted to egg mass was higher for ticks from vaccinated cattle than for ticks from unvaccinated cattle, *M* = 0.517 (51.7%), SD = 0.010 vs. *M* = 0.491 (49.1%), SD = 0.007, respectively, and the difference was significant at *P* = 0.05 (one-tailed two-sample *t*-test, *df* = 4, *P* = 0.0102), (Table 6). There was no statistical difference in the hatching rate between eggs from vaccinated versus unvaccinated cattle, *M* = 0.770 (77.97%), SD = 0.009 vs. *M* = 0.755 (75.53%), SD = 0.020, respectively (one-tailed two-sample *t*-test, *P* = 0.0624) (Table 7), but the hatching rate declined over the course of tick drop, from 78.0% to 66.9% for ticks from control cattle and from 84.0 to 67.7% for vaccinated cattle (Fig. 5).

**Table 5:** Average weight of individual egg masses for a sample of *n* egg masses per day per calf

	Day of collection												
	20		21		22		23		24		25		Mean per calf
	wt	*n*	wt	*n*	wt	*n*	wt	*n*	wt	*n*	wt	*n*	
Control													
C1557	0.146	69	0.150	69	0.131	67	0.136	65	0.116	53	0.100	22	0.130
C1559	0.152	70	0.158	71	0.161	45	0.149	66	0.121	45	0.125	25	0.144
C1560	0.157	71	0.159	69	0.145	67	0.122	68	0.112	49	0.109	11	0.134
Daily mean	0.152		0.156		0.146		0.136		0.116		0.111		0.136
SD	0.0051		0.0049		0.0151		0.0134		0.0048		0.0124		0.007
SEM	0.0030		0.0028		0.0087		0.0077		0.0028		0.0071		0.004
Vaccinated													
C1551	0.159	67	0.168	70	0.163	68	0.160	70	0.154	54	0.154	24	0.160
C1553	0.163	71	0.175	70	0.168	71	0.166	72	0.151	67	0.145	15	0.161
C1554	0.166	69	0.168	68	0.160	68	0.168	67	0.146	59	0.141	25	0.158
Daily mean	0.163		0.171		0.164		0.165		0.150		0.147		0.160
SD	0.0037		0.0040		0.0040		0.0042		0.0041		0.0067		0.0016
SEM	0.0021		0.0023		0.0023		0.0024		0.0024		0.0039		0.0009

wt = Average weight in grams for *n* egg masses from ticks collected on each day. Day of collection = number of days since larval ticks were first applied. *n* = number of ticks include in the average for the day. *SD* standard deviation, *SEM* standard error of the mean

**Table 6:** Conversion rate: average proportion of total body weight converted to eggs for ticks from each calf on each day

	Date of collection												Mean conversion
	20		21		22		23		24		25		
	Rate	*n*	Rate	n	Rate	n	Rate	n	Rate	n	Rate	n	
Control													
C1557	0.499	69	0.511	69	0.472	67	0.515	65	0.474	51	0.454	22	0.488
C1559	0.519	72	0.496	72	0.493	72	0.501	72	0.473	51	0.509	29	0.499
C1560	0.529	72	0.525	72	0.507	72	0.432	72	0.477	53	0.447	16	0.486
Daily mean	0.516		0.511		0.491		0.483		0.475		0.470		0.491
SD	0.0153		0.0145		0.0176		0.0444		0.0021		0.034		0.007
SEM	0.0088		0.0084		0.0102		0.0257		0.0012		0.0196		0.004
Vaccinated													
C1551	0.507	72	0.518	72	0.517	72	0.518	72	0.512	62	0.522	29	0.516
C1553	0.536	72	0.540	72	0.534	72	0.549	72	0.506	71	0.501	15	0.528
C1554	0.529	69	0.507	68	0.508	68	0.506	67	0.489	59	0.506	25	0.508
Daily mean	0.524		0.522		0.520		0.524		0.503		0.501		0.517
SD	0.0149		0.0170		0.0134		0.0220		0.0118		0.0108		0.0102
SEM	0.0086		0.0098		0.0077		0.0127		0.0068		0.0062		0.0059

Average proportion of total body weight converted to eggs for a sample of *n* ticks collected from each calf each day. Day of collection = number of days since larval ticks were first applied. *n* = number of ticks include in the average for the day. *SD* standard deviation, *SEM* standard error of the mean

**Table 7:** Average percent of eggs hatching for a sample of *n* ticks per day per calf

	Day of collection												
	20		21		22		23		24		25		Mean % per calf
	%	*n*	%	*n*	%	*n*	%	*n*	%	*n*	%	*n*	
Control													
C1557	75.25	47	83.50	47	75.06	46	83.09	48	77.39	48	71.88	22	77.70
C1559	78.77	48	77.16	48	78.75	43	68.15	44	76.43	46	63.63	17	73.82
C1560	80.01	48	79.17	45	81.24	46	75.19	46	69.77	47	65.06	11	75.07
Daily mean	78.01		79.94		78.35		75.48		74.53		66.86		75.53
SD	2.46		3.24		3.11		7.47		4.15		4.41		1.98
SEM	1.42		1.87		1.79		4.31		2.40		2.55		1.14
Vaccinated													
C1551	81.72	47	72.36	47	83.20	47	80	47	78.13	46	74.77	24	78.44
C1553	88.29	47	78.31	48	83.30	48	87.92	42	75.90	43	57.55	15	78.55
C1554	84.8	46	81.46	47	78.39	47	79.70	48	66.20	48	70.90	24	76.92
Daily mean	84.95		77.38		81.63		82.69		73.41		67.74		77.97
SD	3.29		4.62		2.81		4.54		6.34		9.04		0.91
SEM	1.90		2.67		1.62		2.62		3.66		5.22		0.53

% = Average percent of a subsample of eggs hatching for a sample of *n* egg masses per day per calf. Day of collection = number of days since larval ticks were first applied. *n* = number of egg masses sampled for the calf on the day. *SD* standard deviation, *SEM* standard error of the mean

### Protein content of replete female ticks

Overall, there was no statistically significant difference in the protein content of ticks (measured as soluble protein as a proportion of total tick weight) from vaccinated versus control cattle, *M* = 0.256 (25.6%), SD = 0.017 vs. *M* = 0.244 (24.4%), SD = 0.006, respectively (one-tailed two-sample *t*-test, *df* = 4, *P* = 0.1538) (Table 8 and Additional file 5: Data file S5).

**Table 8:** Average total soluble protein per gram of tick

	Day of collection						Mean per calf
	20	21	22	23	24	25	
Control							
C1557	0.247	0.249	0.245	0.261	0.232	0.196	0.238
C1559	0.252	0.249	0.244	0.273	0.218	0.222	0.243
C1560	0.245	0.261	0.271	0.253	0.236	0.232	0.250
Daily mean	0.248	0.253	0.253	0.262	0.229	0.217	0.244
SD	0.0036	0.0069	0.0153	0.0101	0.0095	0.0186	0.006
SEM	0.0021	0.0040	0.0088	0.0058	0.0055	0.0107	0.003
Vaccinated							
C1551	0.241	0.233	0.278	0.279	0.223	0.202	0.243
C1553	0.249	0.249	0.291	0.341	0.244	0.277	0.275
C1554	0.232	0.248	0.323	0.272	0.235	0.185	0.249
Daily mean	0.241	0.243	0.297	0.297	0.234	0.221	0.256
SD	0.0085	0.0090	0.0232	0.0380	0.0105	0.0490	0.017
SEM	0.0049	0.0052	0.0134	0.0219	0.0061	0.0283	0.010

Day of collection = number of days since larval ticks were first applied, each value = the average protein content per gram of tick for a random sample of 10 replete ticks per calf per day

## Discussion

We have demonstrated that vaccination of cattle with synthetic peptides corresponding to the predicted extracellular domains of the RmAQP2 protein can produce an immune response that leads to a reduction in the number of ticks successfully feeding to repletion when cattle are challenged with *R. microplus* larvae. There was an overall average of 25% fewer replete female ticks on the vaccinated cattle, all three vaccinated cattle produced fewer ticks than any of the control cattle, but there was substantial variation, and two of the vaccinated cattle (C1553, C1554) performed much better than the third (C1551). Animal health evaluations over the course of the experiment indicated that C1551 suffered from a “failure to thrive” and had slower weight gain than the other cattle, which might explain why this calf was less well protected by vaccination. If we exclude C1551 from the evaluation, there was an overall 32% reduction in ticks produced comparing the three control animals with vaccinated animals C1553 and C1554 alone. However, by all other measures, C1551 performed similarly to the other cattle, and for all further evaluations this calf was considered to be equivalent to the other animals.

Although previously published results from our lab suggested that expression of aquaporin was greatest in the salivary glands [22], a detailed re-examination of the tissue-specific expression of aquaporin 2 at different tick life stages (data not shown) suggested that this gene may be upregulated to a greater extent in the ovary than in the salivary glands. Consequently, we used protein isolated from tick ovaries in protein immunoblots (western blots) to confirm an immune response to native protein. Polyclonal immune sera from vaccinated cattle bound to a ≈50–55 KDa tick ovary protein band on western blots; the same band was absent from either pre-vaccination serum or serum from control animals. These results recapitulate what was seen in the previously published work [22], although using a peptide 2-specific monoclonal antibody binding to native tick gut protein in the previous work allowed bands to be seen much more clearly than we were able to see working with polyclonal serum from vaccinated animals and binding to native ovary protein. Even though clear banding patterns were much more difficult to visualize, the 50–55 KDa band was definitively seen only in vaccinated animals.

Vaccination with synthetic peptides that had been conjugated to KLH and adjuvanted with saponin produced an immune response that was sufficient to impact tick feeding success. For peptides 1 and 3 vaccinated cattle reached the peak of their immune response after the third vaccination with conjugated peptide, the final boost with unconjugated peptides had very little impact on peak titer. In the case peptide 2, all three calves reached their peak titers at week 10, 1 week after the peptide boost. Peptide 1 provided the highest level of response, and there was a similar pattern of response to all peptides across the three vaccinated calves (Figs. 2 and 3). With regard to the overall magnitude of the response, all three cattle responded differently, but consistently, with C1554 achieving the maximum total IgG titers, C1551 intermediate, and C1553 the lowest response. The larval ticks were applied on day 70, 1 week after the final peptide boost at day 63; since titers peaked at 8 weeks (56 days) for peptides 1 and 3 and 10 weeks (70 days) for peptide 2, the larval ticks were first beginning to attach and feed after the highest titers were achieved, and by the time the adults began feeding (20 days after larval ticks were applied) at approximately 85 days after the first vaccination, titers to all three peptides were beginning to drop. Although we could not quantify tick numbers before adult repletion, there appeared to be fewer ticks on the vaccinated animals when we visually inspected the larval ticks in the feeding patch at day 5 and the early nymphal ticks at day 9, suggesting that the survival of larval and nymphal ticks, which were exposed to higher antibody titers than the adults, may have been impacted by vaccination. Days 5 and 9 were chosen for these observations because these time points bracket the time larval ticks would be transitioning to the nymphal stage, a critical point in the development of this one-host tick. We suggest that if the timing of the tick feeding had corresponded better with the peak antibody titers, vaccination may have had a greater impact on the tick survival. As it was, none of the tick life stages, including the adult ticks in particular, which take the largest amount of blood (and thus are exposed to the greatest amount of antibody), were exposed to peak antibody titers. We hypothesize that if we had placed the ticks on the cattle sooner, or continued vaccination longer, they would have been exposed to higher antibody levels, which may have resulted in greater tick mortality. If this is the case, however, in order for these peptides to be an effective vaccine, methods will need to be developed to produce sustained high antibody levels.

The vaccinated calf with the highest overall anti-peptide antibody titers, C1554, produced an intermediate number of replete female ticks compared to the numbers on the other vaccinated calves, which had lower antibody titers, suggesting that there is no direct relationship between titer and vaccine efficacy. Isotyping data showed that C1554 had high levels of both IgG1 and IgG2 but the lowest ratios of IgG1 to IgG2. Ticks were best controlled (fewest replete females) on C1553, which had the lowest overall levels of total IgG antibody but had the highest IgG1/IgG2 ratios, whereas C1551, which had the highest tick burden, had intermediate levels of total IgG and also intermediate IgG1/IgG2 ratios (Table 2). IgG1 and IgG2 are the dominant antibody isotypes in cattle, and each has its own functional phenotype [28]. IgG1 fixes complement [29] and is overwhelmingly preferentially secreted in colostrum [28, 30, 31], whereas IgG2 may be less involved in complement fixation than IgG1 [28, 29] and may be more involved in opsonization [28]. A previous anti-tick vaccine study suggested that levels of IgG1 were correlated with the levels of protection when cattle were vaccinated with tick midgut proteins [32], although that does not appear to be the case in this study. Perhaps because of its role as a secreted component of colostrum [31], IgG1 has become adapted to be functionally more stable outside of the circulatory milieu, and this may make it better able to bind exposed tick aquaporin epitopes once it has passed into the gut of the tick. On the other hand, IgG1 and IgG2 have different sensitivities to proteolysis—for example, in a mixture of IgG1 and IgG2, pepsin will cleave all IgG1 and leave IgG2 intact [28]—and it has been suggested that the protein degradation machinery of the tick might play a role in anti-tick vaccine efficacy [33]. Conceivably, antibody isotypes that are less susceptible to proteolytic degradation might play a more important role in binding to targets in the tick. If IgG2 is involved in opsonization, binding to targets within the tick may promote the activity of the tick innate immune response by activating phagocytic hemocytes. All three vaccinated cattle had high levels of IgG1 but greatly differing levels of IgG2. Levels of IgG2 were lowest in C1551 and C1553, where tick control was the most effective, suggesting that the overall effect is a result of interplay between the isotypes rather than absolute levels of one or the other. Although more data are needed to draw any definitive conclusions, we hypothesize that immune responses that can be directed towards higher IgG1/IgG2 ratios may be more effective. Although Holstein cattle are relatively homogeneous genetically, individual animals will still respond differently to immune stimuli based on their unique immunogenetic backgrounds. It may be that the choice of specific epitopes themselves, or the choice of conjugate or adjuvant, could play some role in directing the antibody isotype responses. It is clear from these data (Figs. 2 and 3, Table 2) that different peptide sequences can stimulate different levels and types of immune responses.

The only treatment difference between control cattle and vaccinated cattle was the presence of the peptides conjugated to the KLH; control cattle were injected with adjuvant and KLH alone (without conjugated peptides). Consequently, any effect on tick survival and feeding success must be related to the immune response to the conjugated peptides and not a result of general upregulation of innate immunity caused by immune stimulation by KLH. KLH has been used previously in anti-tick vaccine research [34] and in other types of vaccine research and development for enhancing immune response to conjugated peptides [35, 36], and although it is highly antigenic, there is little information about how an immune response specific to KLH might impact innate immunity.

The fully fed female ticks dropping from all vaccinated cattle had significantly greater replete weights on average than the controls, which recapitulates the results from the RNA silencing experiments that had previously been performed [22]. We had hypothesized that when the function of RmAQP2 is abrogated, either by RNAi or by binding of immune proteins, ticks may be less able to remove excess water from the blood meal, possibly resulting in reduced levels of protein acquisition and consequent reduced egg production. We tested this hypothesis in two ways, with a protein assay to determine whether the level of soluble protein as a proportion of total tick weight was different in ticks fed on vaccinated versus unvaccinated cattle, and by weighing eggs and calculating the efficiency of conversion of total body weight into weight of eggs. In either case, if the tick weight was greater because excess water was not removed, either the proportion of total body weight converted to eggs or proportion of the total weight made up of soluble protein should be lower for the ticks fed on vaccinated versus control animals, but there was no statistically significant difference overall in either conversion or protein content between ticks fed on vaccinated versus unvaccinated cattle. We conclude that the difference in weight of the blood meals is not related to water content and that the vaccinated ticks were taking larger blood meals.

Interestingly, the difference in weight between ticks fed on vaccinated versus unvaccinated cattle increased with the time it took to feed, with the greatest difference seen in ticks that dropped on the last day (26.25%) as compared to those that dropped on the first day (6.36%), suggesting that the longer the ticks were exposed to the antibody, the greater the effect. Our hypothesis that the differences in replete weight were the result of the inability to remove water from the blood meal was not supported. Since the ticks on vaccinated cattle took longer to feed, the difference in replete weight may be related to feeding time; however, it is unknown how vaccination may influence feeding time. Although we are left with no hypothetical mechanism, it is clear that vaccination has a similar effect to that seen with RNA silencing of aquaporin 2 [22]. Further investigation will be needed to understand the basis of this effect.

Studies on the effect of vaccination on the hatching rate of eggs produced by ticks fed on vaccinated versus control animals confirm that hatching was not impacted by vaccination: the hatching rate was the same for eggs laid by females fed on vaccinated or unvaccinated cattle. Unfortunately, since we weighed total egg production of each female but did not count the number of eggs produced, we cannot draw any conclusion relating to reproductive success of individual female ticks. However, in light of the 25% reduction in the total number of replete females produced, the reproductive success of the population as a whole would have been negatively impacted in the vaccinated group due to the presence of fewer ovipositing females.

Overall, these data suggest that RmAQP2 could be a useful component of an anti-tick vaccine cocktail consisting of multiple targets. Although 25% reduction in the number of replete ticks is significant, it would not be enough by itself to reduce the need to acaricides. It may be necessary to combine several targets to increase the effectiveness of an anti-tick vaccine to levels that will provide sufficient control to do away with the need for acaricide treatment. Multiple targets combined into a single vaccine may also reduce the likelihood that tick populations might adapt and escape control [37, 38]. Most importantly, this work suggests that a full-length expressed protein is not required to induce a protective immune response. Targeting extracellular peptide domains is sufficient to produce an immune response that can interfere with Aquaporin function, resulting in reduced tick survival overall. However, conjugation of peptides to an antigenic carrier such as KLH is critical as we saw that vaccination with peptides alone did not boost titers, even after 3 vaccinations with conjugated peptides. The ability to use peptide domains as vaccine targets may make it easier to include multiple targets to create multivalent vaccines.

There are ongoing research efforts to identify more consistently effective anti-tick vaccine targets; however, there has been little research towards developing methods for maintaining effectively high antibody levels. Since tick gut proteins like the aquaporins are concealed antigens (as are Bm86 and its various orthologs), there is no natural boost from exposure to tick feeding, and consequently methods are needed to maintain high antibody levels in order to get adequate protection without the need for periodic revaccination. Revaccination of animals on a regular basis (as is called for with the Bm86-based vaccines) is expensive and ultimately unsustainable. In order to use anti-tick vaccination approaches targeting concealed antigens in the most effective way, it will be important to explore new methods for expression of antigens to constantly boost antibody production and maintain antibody titers, such as transfection into attenuated persistently infecting pathogen strains, as has been suggested for *Babesia bovis* [39, 40].

## Conclusions

Vaccination resulted in a 25% reduction in the number of ticks feeding to repletion. Although this is not sufficient to eliminate the need for acaricides, it does suggest that RmAQP2 could be a useful component of an anti-tick vaccine cocktail which includes other antigens. Further, we have shown here that peptide vaccination can be an effective tool for testing antigens, bypassing the need for expression of full-length protein targets.

## Supplementary Information

**Additional file 1.** Timecourse of IgG antibody levels  at a 1 to 256 dilution.

**Additional file 2.** Peak IgG antibody titer.

**Additional file 3.** IgG1-IgG2 antibody isotyping.

**Additional file 4.** Data for all individual ticks.

**Additional file 5.** Protein assay.

## Data Availability

All relevant raw data used to support the conclusions of this study have been included as additional data files or are available from the corresponding author.

## Abbreviations

Bm86: *Boophilus microplus* antigen 86
BSA: Bovine serum albumin
*df*: Degrees of freedom
DMSO: Dimethyl sulfoxide
ELISA: Enzyme-linked immunosorbent assay
IgG: Immunoglobulin G
IgG1: Immunoglobulin G subclass 1
IgG2: Immunoglobulin G subclass 2
KDa: Kilodaltons
KLH: Keyhole limpet hemocyanin
M: Mean
PBS: Phosphate-buffered saline
RmAQP2: *R. microplus* aquaporin 2
SD: Standard deviation
